# The Association Between Stress‐Induced Hyperglycemia Ratio and Increased Urinary Albumin Excretion in Patients With Hypertension: A Population‐Based Study

**DOI:** 10.1002/kjm2.70237

**Published:** 2026-05-14

**Authors:** Kai‐Jun Zhang, Yun Lan, Wen‐Feng Zeng, Rong‐Ting Zhang, Zhi‐Yi Ma, Xin Zou

**Affiliations:** ^1^ Department of Pulmonary and Critical Care Medicine Longyan First Affiliated Hospital of Fujian Medical University Longyan China; ^2^ Department of Rehabilitation Xinqiao Health Center, Changting County Longyan China; ^3^ State Key Laboratory of Cardiovascular Diseases and Medical Innovation Center Shanghai East Hospital, School of Medicine, Tongji University Shanghai China

**Keywords:** albuminuria, hypertension, NHANES, renal function, stress hyperglycemia ratio

## Abstract

The stress hyperglycemia ratio (SHR), a novel marker reflecting relative hyperglycemia, has been increasingly recognized for its prognostic value in cardiovascular and metabolic diseases. However, its association with early renal damage in hypertensive patients remains underexplored. This study aims to investigate the independent relationship between SHR and albuminuria in a nationally representative cohort of adults with hypertension. This cross‐sectional analysis included 8732 adults with hypertension from the National Health and Nutrition Examination Survey 2005–2018. Multivariable logistic regression models were used to evaluate the independent association between SHR and albuminuria, with adjustment for demographic, lifestyle, and clinical covariates. Restricted cubic splines (RCS) were employed to model nonlinear relationships. Stratified analyses were conducted across various subgroups. The prevalence of albuminuria was 15%. After full adjustment for confounders, compared to the reference quartile (Q2), the highest SHR quartile was significantly associated with an increased odds of albuminuria (OR: 2.34; 95% CI: 1.62–3.36; *p* < 0.001). RCS analysis revealed a nonlinear, J‐shaped association between continuous SHR and albuminuria (*p* for nonlinearity < 0.001), with risk markedly increasing beyond an SHR of approximately 0.91. Subgroup analyses confirmed the robustness of this association across most strata, including age, gender, BMI, eGFR, diabetes status, and cancer history, with no significant interactions observed (all *p*‐interaction > 0.05). In this large, population‐based study, an elevated SHR is significantly and independently associated with a higher prevalence of albuminuria among US adults with hypertension.

**Abbreviations**
BMI: body mass index
CKD: chronic kidney disease
CVD: cardiovascular disease
DBP: diastolic blood pressure
eGFR: estimated glomerular filtration rate
FBG: fasting blood glucose
HbA1c: hemoglobin A1c
NCHS: National Center for Health Statistics
NHANES: National Health and Nutrition Examination Survey
PIR: poverty income ratio
RCS: restricted cubic spline
SBP: systolic blood pressure
SHR: stress hyperglycemia ratio
UACR: urinary albumin‐to‐creatinine ratio

## 1 Introduction

Hypertension remains a paramount public health challenge and a leading contributor to global morbidity and mortality, primarily through its devastating effects on target organs, including the heart, brain, and kidneys [1, 2]. The renal system is both a target and a contributor to hypertensive damage, with the progression of renal impairment often being insidious [3]. Albuminuria, clinically manifested as an abnormally high urinary albumin excretion, is a well‐established early marker of glomerular endothelial dysfunction and renal damage [4, 5]. The presence of albuminuria not only signifies an increased risk for the progression to overt diabetic and nondiabetic chronic kidney disease (CKD) but also serves as a powerful independent predictor of cardiovascular events and all‐cause mortality [6, 7, 8]. Therefore, identifying modifiable risk factors and novel biomarkers that can detect hypertensive individuals at the highest risk for incipient renal damage is of critical clinical importance.

Traditionally, dysglycemia, particularly in the context of diabetes mellitus, has been inextricably linked to the development and progression of nephropathy [9, 10]. The assessment of chronic glycemic control via glycated hemoglobin (HbA1c) is a cornerstone of risk prediction [11]. However, a growing body of evidence suggests that acute, transient spikes in blood glucose—often triggered by physiological stress—may inflict distinct and potent detrimental effects on the vascular endothelium, independently of chronic hyperglycemia [12, 13]. These glycemic fluctuations can induce oxidative stress, activate inflammatory pathways, and cause endothelial dysfunction more aggressively than sustained hyperglycemia [14]. Measuring these acute surges in routine clinical practice has been challenging.

The stress hyperglycemia ratio (SHR) has recently emerged as an innovative metric to quantify this phenomenon. It is calculated as the ratio of fasting blood glucose (FBG) to HbA1c [15, 16]. By factoring out the underlying chronic glycemic burden reflected by HbA1c, SHR aims to isolate the component of hyperglycemia attributable to an acute stress response [17]. Initially studied in critical care and acute myocardial infarction settings, where it proved to be a superior predictor of mortality than admission glucose alone [18, 19], SHR's role in chronic conditions is now being unraveled. Elevated SHR has been associated with poor outcomes in heart failure, stroke, and in patients undergoing percutaneous coronary intervention [20, 21, 22]. The pathophysiological mechanisms may involve endothelial dysfunction, increased oxidative stress, and a heightened inflammatory state induced by acute glucose fluctuations, all of which are also central to the development and progression of renal microvascular damage and albuminuria [23].

Despite the clear pathophysiological parallels, the relationship between SHR and early renal damage, specifically in a hypertensive population, has not been thoroughly investigated. Hypertensive individuals already endure a heightened state of vascular stress; superimposing acute relative hyperglycemia could potentially accelerate target organ damage, including in the kidneys. Understanding this relationship could unveil SHR as a simple, inexpensive, and effective tool for risk stratification in routine clinical practice. Therefore, utilizing data from the National Health and Nutrition Examination Survey (NHANES), a large, nationally representative database, this study aimed to: (1) Examine the independent association between SHR and the prevalence of albuminuria in adults with hypertension; (2) Explore the shape of the dose–response relationship using restricted cubic splines; (3) Investigate the consistency of this association across key demographic and clinical subgroups.

## 2 Methods

### 2.1 Study Population and Design

This study utilized data from seven continuous cycles of the NHANES (2005–2006, 2007–2008, 2009–2010, 2011–2012, 2013–2014, 2015–2016, and 2017–2018). NHANES is a complex, stratified, multistage probability survey conducted by the National Center for Health Statistics (NCHS) to assess the health and nutritional status of the non‐institutionalized civilian population in the United States. The survey protocol was approved by the NCHS Research Ethics Review Board, and all participants provided written informed consent.

**FIGURE 1:**
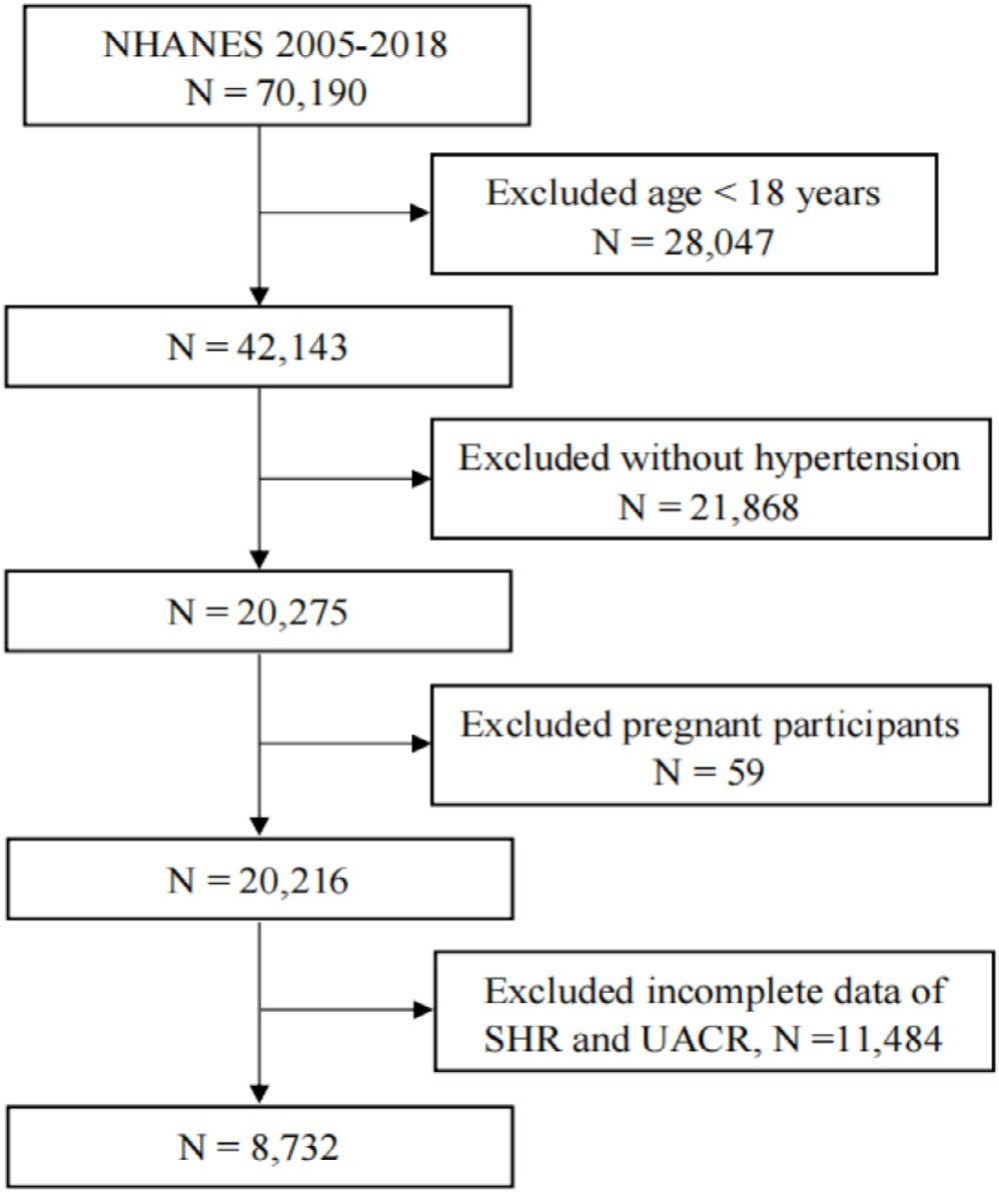
Flow chart of study population.

The initial pool comprised 70,190 participants across the seven cycles. We sequentially applied the following exclusion criteria (Figure 1). Participants aged below 18 years (*N* = 28,047) were excluded. From the remaining 42,143 adults, those without hypertension (*N* = 21,868) were excluded. Consistent with the 2017 ACC/AHA hypertension guidelines, hypertension was defined by the presence of any of the following criteria: (a) self‐reported diagnosis by a physician; (b) self‐reported current use of antihypertensive medication; or (c) average systolic blood pressure ≥ 130 mmHg or diastolic blood pressure ≥ 80 mmHg from three mobile examination center (MEC) measurements. Pregnant women (*N* = 59) were excluded. And participants with missing data on SHR or urinary albumin‐to‐creatinine ratio (UACR) (*N* = 11,484) were excluded. The final analytical sample consisted of 8732 participants with complete data.

### 2.2 Definition of SHR and Albuminuria

The SHR was calculated using the formula: [FBG (mmol/L)]/[1.59 * HbA1c (%) − 2.59] [15]. For analysis, SHR was evaluated both as a continuous variable and categorized into quartiles (Q1–Q4) based on its distribution within the study population. Albuminuria was defined as a UACR ≥ 30 mg/g, in accordance with the Kidney Disease: Improving Global Outcomes (KDIGO) guidelines [24].

### 2.3 Assessment of Covariates

Comprehensive data on demographic, lifestyle, and clinical factors were collected through household interviews, questionnaires, and standardized physical examinations in the MEC. Demographic factors: Age, sex, race/ethnicity (categorized as Mexican American, Non‐Hispanic White, Non‐Hispanic Black, Other), educational attainment (< 12th grade, 12th grade, > 12th grade), marital status, and poverty income ratio (PIR). Lifestyle factors: Smoking status (never, former, current), alcohol drinking status (yes/no), and physical activity level (categorized based on self‐reported frequency and intensity into less than moderate, moderate, and vigorous). Anthropometric and clinical measurements: Body mass index (BMI), systolic blood pressure (SBP), and diastolic blood pressure (DBP). Laboratory measurements included total cholesterol, uric acid, serum hemoglobin, and estimated glomerular filtration rate (eGFR), calculated using the CKD‐EPI equation [25]. Comorbidities: History of diabetes, cardiovascular disease (CVD), and cancer were based on self‐reported physician diagnosis.

### 2.4 Statistical Analysis

Baseline characteristics across SHR quartiles were presented as mean with standard deviation or median with interquartile range for continuous variables and percentages (numbers) for categorical variables. One‐way analysis of variance (ANOVA) (for continuous variables) and chi‐square tests (for categorical variables) were used to compare differences across quartiles.

The association between SHR and albuminuria was evaluated using three weighted multivariable logistic regression models. Model 1: Unadjusted. Model 2: Adjusted for age, sex, and race/ethnicity. Model 3: Fully adjusted for age, sex, race/ethnicity, BMI, education, PIR, smoking status, drinking status, physical activity, marital status, total cholesterol, uric acid, hemoglobin, eGFR, diabetes, CVD, and cancer. SHR was analyzed both as a continuous variable and as quartiles (with Quartile 2 as the reference group due to its lowest risk position in initial analyses). Multicollinearity was evaluated using variance inflation factors (VIF); all values were below 5.0 (Table S1), indicating no significant collinearity among the covariates and ensuring the reliability of the regression estimates. To explore the potential nonlinear relationship between SHR and albuminuria, we performed a restricted cubic spline (RCS) analysis with 4 knots placed at the 5th, 35th, 65th, and 95th percentiles. The likelihood ratio test was used to assess overall significance and nonlinearity. To evaluate the consistency of the association, we conducted stratified analyses by pre‐specified subgroups: age (< 65 vs. ≥ 65 years), sex, BMI (< 30 vs. ≥ 30 kg/m^2^), eGFR (< 60 vs. ≥ 60 mL/min/1.73m^2^), diabetes status, and cancer history. Interaction terms between SHR and subgroup variables were introduced into the fully adjusted model to test for effect modification. To address potential confounding by inflammation and insulin resistance, we performed two sensitivity analyses. First, we adjusted for white blood cell (WBC) count as a proxy for systemic inflammation. Second, we adjusted for the triglyceride‐glucose (TyG) index—calculated as Ln[fasting triglyceride (mg/dL) × fasting glucose (mg/dL)/2]—which serves as a surrogate for insulin resistance.

All statistical analyses were performed using R software (version 4.1.3, The R Foundation). A two‐sided *p*‐value < 0.05 was considered statistically significant.

## 3 Results

### 3.1 Baseline Characteristics of the Study Population

The weighted baseline characteristics of the overall population and by SHR quartiles are detailed in Table 1. The mean age of the participants was 55 (±16) years, 52% were male, and 68% were Non‐Hispanic White. The mean SHR was 0.94 (±0.14). There were significant differences across SHR quartiles for most variables. Participants in the highest SHR quartile (Q4) were more likely to be male. They had significantly higher levels of FBG, uric acid, hemoglobin, and a markedly higher prevalence of diabetes (36% in Q4 vs. 23% in Q1 and 12% in Q2). The overall prevalence of albuminuria was 15%. Q2 exhibited the lowest albuminuria rate at 11%, and was thus chosen as the reference group. While the prevalence of albuminuria in the Q4 group was the highest at 20%. The absolute difference in albuminuria prevalence between Q2 and Q4 was 9.0% (11.0% vs. 20.0%).

**TABLE 1:** Baseline characteristics of study population according to SHR quartiles (weighted).

Characteristic	Overall	Quartile 1	Quartile 2	Quartile 3	Quartile 4	*p*
Age (year)	55 (16)	56 (16)	56 (15)	54 (15)	54 (16)	< 0.001
Gender (%)						< 0.001
Male	4563 (52%)	1150 (41%)	1008 (46%)	1105 (58%)	1300 (63%)	
Female	4169 (48%)	1478 (59%)	1065 (54%)	839 (42%)	787 (37%)	
Race (%)						< 0.001
Mexican American	1119 (6.5%)	292 (6.1%)	255 (6.4%)	270 (6.5%)	302 (7.1%)	
Non‐Hispanic White	3760 (68%)	876 (57%)	928 (69%)	955 (73%)	1001 (73%)	
Non‐Hispanic Black	2185 (14%)	981 (25%)	463 (12%)	335 (8.7%)	406 (9.4%)	
Other	1668 (12%)	479 (12%)	427 (13%)	384 (12%)	378 (10%)	
Educational attainment (%)						0.002
< 12	2358 (18%)	754 (21%)	506 (16%)	521 (18%)	577 (17%)	
12	2142 (26%)	674 (27%)	529 (28%)	447 (24%)	492 (25%)	
> 12	4114 (56%)	1165 (52%)	1013 (56%)	959 (58%)	977 (58%)	
Marital status (%)						0.6
Married/living with partner	5188 (65%)	1488 (63%)	1249 (65%)	1199 (65%)	1252 (65%)	
Without partner	3457 (35%)	1114 (37%)	806 (35%)	733 (35%)	804 (35%)	
Poverty income ratio (%)						< 0.001
< 1.3	2558 (22%)	827 (25%)	595 (21%)	526 (19%)	610 (21%)	
1.3–3.5	3079 (38%)	954 (41%)	696 (36%)	680 (38%)	749 (37%)	
> 3.5	2271 (41%)	604 (34%)	575 (43%)	551 (43%)	541 (42%)	
BMI (kg/m^2^)	31 (7)	31 (8)	30 (7)	31 (7)	32 (7)	0.002
SBP (mmHg)	131 (17)	132 (18)	131 (17)	130 (17)	132 (17)	0.016
DBP (mmHg)	73 (14)	71 (15)	74 (13)	74 (13)	75 (14)	< 0.001
Smoking status (%)						0.003
Never smoker	4429 (51%)	1352 (50%)	1084 (52%)	986 (51%)	1007 (50%)	
Former smoker	2535 (30%)	696 (27%)	560 (28%)	574 (32%)	705 (33%)	
Current smoker	1690 (19%)	559 (22%)	410 (20%)	374 (17%)	347 (16%)	
Physical activity (%)						0.3
Less than moderate	4580 (53%)	1415 (56%)	1060 (53%)	999 (53%)	1106 (52%)	
Moderate	2098 (30%)	652 (29%)	523 (32%)	456 (29%)	467 (29%)	
Vigorous	1100 (17%)	300 (15%)	268 (16%)	270 (18%)	262 (19%)	
Drinking (%)	4689 (74%)	1317 (66%)	1116 (72%)	1097 (77%)	1159 (79%)	< 0.001
Hemoglobin A1c (%)	5.86 (1.10)	6.07 (1.03)	5.75 (0.71)	5.71 (0.92)	5.91 (1.53)	< 0.001
FBG (mmol/L)	6.31 (2.06)	5.50 (1.11)	5.84 (1.02)	6.26 (1.40)	7.65 (3.17)	< 0.001
Total cholesterol (mg/dL)	196 (43)	196 (45)	201 (43)	194 (41)	193 (44)	< 0.001
Uric acid (mg/dL)	5.82 (1.46)	5.75 (1.53)	5.72 (1.42)	5.84 (1.36)	5.97 (1.49)	0.001
Hemoglobin (g/dL)	14.35 (1.53)	13.83 (1.54)	14.22 (1.44)	14.60 (1.46)	14.74 (1.52)	< 0.001
eGFR (ml/min/1.73 m^2^)	88 (22)	86 (24)	87 (22)	89 (21)	89 (22)	< 0.001
Diabetes (%)	2473 (22%)	722 (23%)	339 (12%)	431 (18%)	981 (36%)	< 0.001
CVD (%)	1564 (15%)	496 (17%)	354 (14%)	332 (15%)	382 (15%)	0.2
Cancer (%)	1108 (13%)	342 (15%)	272 (14%)	247 (13%)	247 (12%)	0.4
SHR	0.94 (0.14)	0.79 (0.07)	0.89 (0.02)	0.96 (0.02)	1.11 (0.12)	< 0.001
Albuminuria (%)	1692 (15%)	516 (16%)	330 (11%)	330 (13%)	516 (20%)	< 0.001

Abbreviations: BMI, body mass index; CVD, cardiovascular disease; DBP, diastolic blood pressure; eGFR, estimated glomerular filtration rate; FBG, fasting blood glucose; SBP, systolic blood pressure; SHR, stress hyperglycemia ratio.

### 3.2 Association Between SHR and Albuminuria

The results of the logistic regression analyses are presented in Table 2. In the unadjusted model (Model 1), each unit increase in SHR (continuous) was associated with a 2.83–fold increased odds of albuminuria (OR: 2.83, 95% CI: 1.69–4.73). When analyzed by quartiles, compared to Q2 (reference), Q1 (OR: 1.60, 95% CI: 1.29–2.00), Q3 (OR: 1.26, 95% CI: 0.98–1.60), and Q4 (OR: 2.03, 95% CI: 1.61–2.54) all showed elevated odds, with a significant positive trend (*p*‐trend < 0.001).

**TABLE 2:** Logistic regression analysis of the association between SHR and albuminuria.

Categories	Model 1		Model 2		Model 3	
	OR (95% CI)	*p*	OR (95% CI)	*p*	OR (95% CI)	*p*
Continuous	2.83 (1.69–4.73)	< 0.001	4.47 (2.56–7.81)	< 0.001	3.75 (2.06–6.85)	< 0.001
Categories						
Quartile 1	1.60 (1.29–2.00)	< 0.001	1.46 (1.15–1.85)	0.002	1.46 (1.06–2.02)	0.022
Quartile 2	Ref		Ref		Ref	
Quartile 3	1.26 (0.98–1.60)	0.068	1.38 (1.07–1.76)	0.012	1.47 (1.04–2.08)	0.028
Quartile 4	2.03 (1.61–2.54)	< 0.001	2.29 (1.79–2.91)	< 0.001	2.34 (1.62–3.36)	< 0.001
*p* for trend		< 0.001		< 0.001		< 0.001

*Note:* Model 1: unadjusted. Model 2: adjusted for age, sex, race. Model 3: adjusted for age, sex, race, body mass index, education, poverty income ratio, smoking status, drinking status, physical activity, marital status, total cholesterol, uric acid, hemoglobin, estimated glomerular filtration rate, diabetes, cardiovascular disease, and cancer.

After adjusting for age, sex, and race (Model 2), the associations were strengthened for the continuous SHR (OR: 4.47, 95% CI: 2.56–7.81) and the quartiles (Q4 OR: 2.29, 95% CI: 1.79–2.91). In the fully adjusted model (Model 3), which included socioeconomic, lifestyle, biochemical, and comorbidity factors, the association remained robust and statistically significant. Per unit increase in SHR was associated with a 3.75‐fold higher odds of albuminuria (OR: 3.75, 95% CI: 2.06–6.85). Compared to Q2, the odds ratios for albuminuria were 1.46 (95% CI: 1.06–2.02) for Q1, 1.47 (95% CI: 1.04–2.08) for Q3, and 2.34 (95% CI: 1.62–3.36) for Q4. The *p*‐value for trend across quartiles was highly significant (< 0.001), indicating a clear dose–response relationship.

### 3.3 Nonlinear Relationship Analysis

**FIGURE 2:**
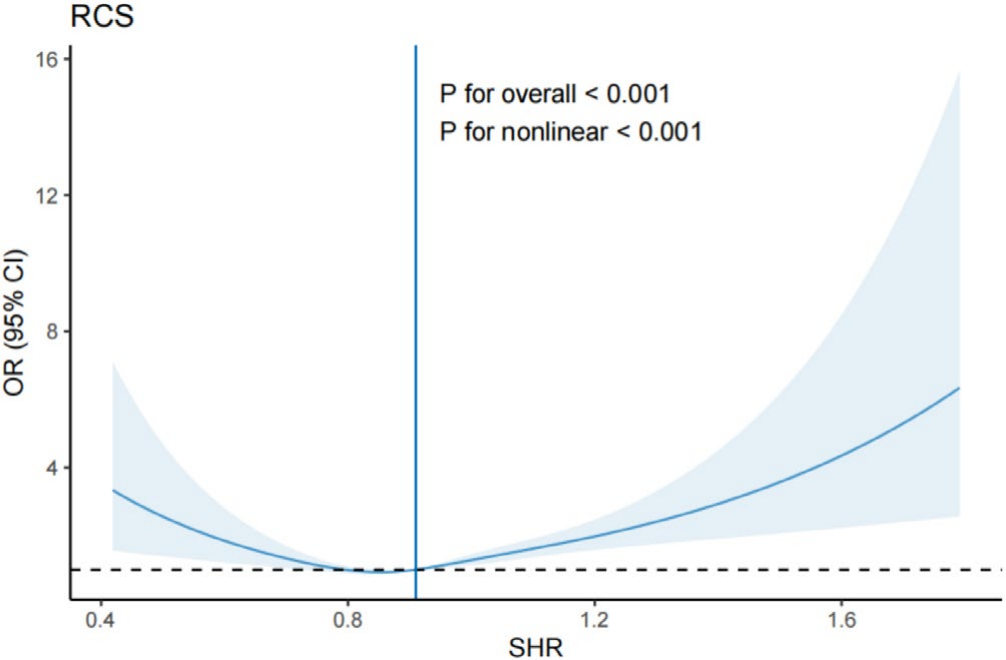
Restricted cubic spline analysis of the association between SHR and increased urinary albumin excretion in patients with hypertension. Adjustment factors included age, sex, race/ethnicity, BMI, education, PIR, smoking status, drinking status, physical activity, marital status, total cholesterol, uric acid, hemoglobin, eGFR, diabetes, CVD, and cancer. CI, confidence interval; OR, odds ratio; SHR, stress hyperglycemia ratio.

The restricted cubic spline analysis (Figure 2) confirmed a significant nonlinear, J‐shaped relationship between SHR and albuminuria after full adjustment (*p* for overall association < 0.001, *p* for nonlinearity < 0.001). The risk of albuminuria was elevated at the lower end of the SHR spectrum (left limb of the “J”), decreased to a nadir around an SHR of 0.87 (corresponding to the reference Q2), and then rose steeply with increasing SHR values beyond approximately 0.91. This curve visually reinforces the quartile findings, explaining the elevated risk in both Q1 and Q4.

### 3.4 Subgroup Analyses

**FIGURE 3:**
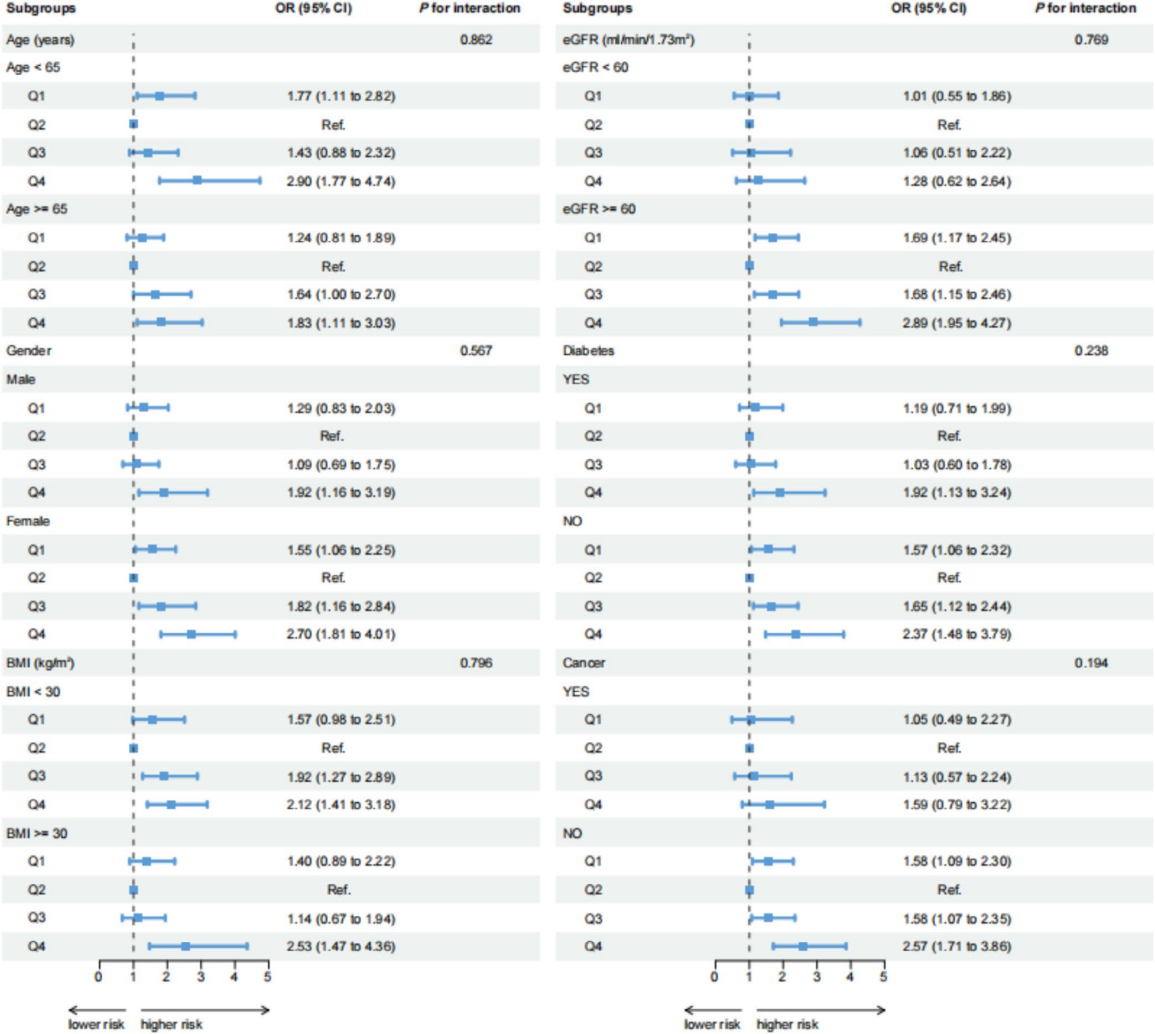
Forest plot of subgroup analyses for the association between SHR and increased urinary albumin excretion in adults with hypertension. Each subgroup analysis adjusted for age, sex, race/ethnicity, BMI, education, PIR, smoking status, drinking status, physical activity, marital status, total cholesterol, uric acid, hemoglobin, eGFR, diabetes, CVD, and cancer, except for the stratifying variable BMI, body mass index; CI, confidence interval; eGFR, estimated glomerular filtration rate; OR, odds ratio; SHR, stress hyperglycemia ratio.

Stratified analyses were performed to assess the consistency of the association between SHR quartiles and albuminuria across various patient subgroups (Figure 3). The positive association between higher SHR quartiles (particularly Q4) and increased odds of albuminuria was generally consistent across all subgroups, including age, gender, BMI, renal function (eGFR), diabetes status, and cancer history.

Notably, the point estimates for Q4 were elevated in nearly every stratum. Formal tests for interaction were not statistically significant for any of the subgroups (all *p*‐interaction > 0.05), indicating that the effect of SHR on albuminuria did not significantly differ by age, gender, BMI, diabetes status, or cancer history. Although the point estimates for Q4 appeared higher in participants with eGFR ≥ 60 (OR: 2.89) compared to those with eGFR < 60 (OR: 1.28), the interaction *p*‐value (0.769) was not significant, suggesting that the difference may be due to chance or reduced power in the smaller CKD subgroup. Similarly, the association seemed pronounced in both diabetes (Q4 OR: 1.92) and non‐diabetes (Q4 OR: 2.37) with no significant interaction (*p* = 0.238), underscoring that the effect of SHR is independent of underlying diabetes status.

### 3.5 Multivariate Model of Albuminuria Risk Factors

Table 3 presents the full results of the multivariate logistic regression model (Model 3) showing the adjusted odds ratios for all covariates included in the analysis alongside SHR quartiles. Besides SHR, older age, current smoking, higher total cholesterol, higher uric acid, lower hemoglobin, lower eGFR, history of diabetes, and history of CVD were independently associated with increased odds of albuminuria. Moderate and vigorous physical activity were protective factors. This model confirms that the association between SHR and albuminuria is independent of these extensive potential confounders.

**TABLE 3:** Multivariate logistic regression models of albuminuria.

Characteristic	OR (95% CI)	*p*
SHR quartiles		
Quartile 1	1.46 (1.06, 2.02)	0.022
Quartile 2	Ref	
Quartile 3	1.47 (1.04, 2.08)	0.028
Quartile 4	2.34 (1.62, 3.36)	< 0.001
Age (year)	1.02 (1.00, 1.03)	0.014
Gender	0.86 (0.63, 1.18)	0.343
Race		
Mexican American	Ref	
Non‐Hispanic White	0.46 (0.33, 0.63)	< 0.001
Non‐Hispanic Black	0.61 (0.45, 0.82)	0.001
Other	0.68 (0.50, 0.93)	0.015
BMI (kg/m^2^)	1.01 (0.99, 1.02)	0.232
Education		
< 12	Ref	
12	0.98 (0.74, 1.28)	0.868
> 12	0.75 (0.53, 1.05)	0.094
Smoking status		
Never smoker	Ref	
Former smoker	1.14 (0.90, 1.43)	0.266
Current smoker	1.54 (1.09, 2.19)	0.016
Drinking	0.81 (0.64, 1.02)	0.067
Poverty income ratio		
< 1.3	Ref	
1.3–3.5	0.98 (0.78, 1.24)	0.864
> 3.5	0.73 (0.53, 1.01)	0.057
Physical activity		
Less than moderate	Ref	
Moderate	0.71 (0.54, 0.94)	0.017
Vigorous	0.54 (0.35, 0.84)	0.007
Marital status		
Married/living with partner	Ref	
Without partner	1.24 (1.00, 1.54)	0.053
Total cholesterol (mg/dL)	1.00 (1.00, 1.01)	< 0.001
Uric acid (mg/dL)	1.10 (1.02, 1.20)	0.022
Hemoglobin (g/dL)	0.86 (0.79, 0.94)	< 0.001
eGFR (ml/min/1.73 m^2^)	0.99 (0.98, 1.00)	0.038
Diabetes	2.29 (1.82, 2.89)	< 0.001
CVD	1.70 (1.36, 2.13)	< 0.001
Cancer	1.05 (0.73, 1.49)	0.804

Abbreviations: BMI, body mass index; CVD, cardiovascular disease; eGFR, estimated glomerular filtration rate; SHR, stress hyperglycemia ratio.

### 3.6 Sensitivity Analyses

In sensitivity analyses, the independent association between SHR and albuminuria persisted after further adjustment for inflammatory and metabolic markers. When incorporating WBC count into the fully adjusted model, the OR for the highest SHR quartile was 2.34 (95% CI: 1.63–3.37) (Table S2). Similarly, adjusting for the TyG index did not significantly alter the findings (OR: 2.23; 95% CI: 1.52–3.26) (Table S3).

In a sensitivity analysis using the lowest quartile (Q1) as the reference, the association between the highest SHR quartile and albuminuria remained robust (OR: 1.60, 95% CI: 1.22–2.09). While Q2 was associated with a significantly lower odds of albuminuria (OR: 0.68, 95% CI: 0.49–0.94), confirming the J‐shaped nature of the association (Table S4).

## 4 Discussion

This large, cross‐sectional, population‐based study provides compelling evidence that an elevated stress hyperglycemia ratio is independently associated with a higher prevalence of albuminuria among US adults with hypertension. Several key findings emerge from our analysis. First, after comprehensive adjustment for a wide array of potential confounders, including the crucial presence of diabetes itself, SHR remained a strong, independent risk factor for albuminuria. Second, we identified a significant nonlinear, J‐shaped dose–response relationship, with the risk of albuminuria escalating sharply beyond a certain SHR threshold. Third, this association was robust and consistent across major demographic and clinical subgroups, including participants with and without diabetes, indicating that the effect of relative hyperglycemia on renal microvasculature is pervasive. These findings suggest that SHR, a simple metric derived from routine laboratory tests, may be a novel and valuable biomarker for identifying hypertensive patients at heightened risk for subclinical renal damage.

The strength of the association is notable. Participants in the highest SHR quartile had a 2.34–fold increased odds of albuminuria compared to those in the reference quartile, even after accounting for absolute chronic glycemic levels (via diabetes status). This suggests that the acute relative hyperglycemic state captured by SHR imposes an additional, distinct risk on renal endothelial function beyond that of chronic hyperglycemia. Acute glucose fluctuations are known to induce more pronounced oxidative stress and provoke a stronger inflammatory response than sustained chronic hyperglycemia [12, 13]. These processes lead to endothelial dysfunction, a reduction in nitric oxide bioavailability, and increased vascular permeability, which are direct precursors to albumin leakage into the urine [23]. In hypertensive individuals, whose vasculature is already under pressure and may have impaired compensatory mechanisms, this acute metabolic stressor could be particularly deleterious, accelerating glomerular damage.

The J‐shaped curve we observed is a fascinating and clinically relevant result. The elevated risk in the highest quartile (Q4) aligns with the hypothesis that acute hyperglycemic stress, independent of chronic glycemic control, is detrimental to renal microvasculature. Proposed mechanisms include: (1) Oxidative Stress: Acute glucose spikes potently generate reactive oxygen species, overwhelming antioxidant defenses and leading to oxidative damage in endothelial cells and podocytes [14, 26]. (2) Inflammation: Transient hyperglycemia can activate key pro‐inflammatory pathways, such as nuclear factor kappa‐B (NF‐κB), increasing the expression of adhesion molecules and cytokines like IL‐6 and TNF‐α, which promote endothelial dysfunction and albumin leakage [27, 28]. (3) Endothelial Dysfunction: Hyperglycemia impairs endothelium‐dependent vasodilation, partly by reducing the bioavailability of nitric oxide, which is crucial for maintaining glomerular hemodynamics and barrier function [29]. The kidney's glomeruli, with their high flow and pressure, are especially susceptible to this endothelial insult. However, after separate adjustment for WBC count and TyG index, the independent association between elevated SHR and albuminuria remained significant, suggesting that inflammation and insulin resistance alone do not fully explain the observed relationship. Furthermore, the persistence of the association after adjusting for these surrogates does not rule out partial mediation—rather, it suggests that other pathways (e.g., direct endothelial dysfunction, oxidative stress, or vascular permeability) may also contribute. Nevertheless, these surrogates are indirect; direct measurements of endothelial nitric oxide synthase activity, circulating adhesion molecules, or vascular permeability are not available in NHANES. Future studies incorporating such direct markers are needed to confirm the hypothesized mechanisms.

The significantly elevated risk in the lowest SHR quartile (Q1) was unexpected and warrants careful interpretation. This group likely comprises two distinct populations: individuals with excellent glycemic control (low FBG and low HbA1c) and individuals with discordantly low FBG relative to their HbA1c, which could indicate episodes of hypoglycemia, liver disease, or other conditions affecting glucose homeostasis [30, 31]. Hypoglycemia itself is a potent physiological stressor that can provoke a counter‐regulatory hormone response, leading to hemodynamic changes, increased oxidative stress, and inflammation [32]. This counter‐regulatory surge could theoretically induce renal vascular stress and contribute to albuminuria. Furthermore, hypoglycemia is associated with hypotension and reduced renal perfusion in susceptible individuals, which might cause tubular damage and impair albumin reabsorption [33]. Although the RCS analysis suggested a J‐shaped association between SHR and albuminuria, the mechanisms underlying the increased risk at lower SHR levels cannot be determined in this cross‐sectional study. Potential contributors such as liver dysfunction, frailty, or malnutrition. In addition, the potential influence of glucose‐lowering medications should be considered. Intensive glycemic control or certain antidiabetic therapies may predispose individuals to hypoglycemic episodes or increased glycemic variability, which could contribute to renal hemodynamic instability and endothelial dysfunction. Nevertheless, consistent with recent systematic evidence [34], our findings add to the growing evidence that SHR may display a U‐shaped or J‐shaped relationship with adverse health outcomes.

While the RCS curve suggests an inflection point at approximately 0.91, this value should not be interpreted as a universal clinical treatment threshold. Rather, it should be regarded as a study‐specific risk stratification point. Prospective longitudinal studies are needed to determine whether this threshold has clinical utility and to validate its potential role in guiding intervention.

A critical aspect of our study is the demonstration that the SHR‐albuminuria association is independent of diabetes status. The stratified analysis confirmed that both non‐diabetic and diabetic hypertensive individuals with high SHR are at increased risk. This is clinically significant because it suggests that screening for relative hyperglycemia could be beneficial even in non‐diabetic hypertensive patients, a group not traditionally targeted for intensive glucose monitoring. SHR could help identify a subset of “metabolically vulnerable” hypertensives who are at stealth risk for renal impairment despite not having crossed the threshold for diabetes diagnosis. The consistency of the association across subgroups reinforces its generalizability.

Although no statistically significant interactions were detected in the stratified analyses (all *p*‐interaction > 0.05), the point estimates for the association between elevated SHR and albuminuria were numerically more pronounced in females, participants with obesity (BMI ≥ 30 kg/m^2^), and those with preserved renal function (eGFR ≥ 60 mL/min/1.73 m^2^). These observations should be interpreted with caution and are strictly hypothesis‐generating. Regarding sex‐specific differences, estrogen has been shown to modulate both the hypothalamic–pituitary–adrenal axis and the sympathetic nervous system response to stress, which could potentially amplify the hemodynamic and glucoregulatory consequences of acute hyperglycemia in women [35]. Additionally, obesity is characterized by a state of chronic low‐grade inflammation and adipokine dysregulation, which may sensitize the renal microvasculature to the acute deleterious effects of stress‐induced hyperglycemia, thereby augmenting glomerular hyperfiltration and albumin leakage [36]. The more prominent OR in those with eGFR ≥ 60 mL/min/1.73 m^2^ may reflect the sensitivity of albuminuria as an early marker of kidney damage. In patients with relatively preserved kidney function, SHR might be a more potent driver of “early‐stage” glomerular hyperfiltration and permeability changes compared to those with advanced chronic kidney disease, where multiple competing risk factors are already present.

The implications of our study are twofold. From a pathophysiological perspective, it adds to the growing evidence that glycemic variability and acute hyperglycemic spikes are active players in vascular damage, not merely innocent bystanders [37]. It shifts the focus from average chronic glucose levels to the stability of the glycemic environment. From a clinical perspective, SHR is easily calculable from routine lab work (FBG and HbA1c) [38]. Incorporating this ratio into risk assessment could help clinicians identify hypertensive patients who, despite seemingly acceptable HbA1c levels, are experiencing harmful glycemic fluctuations and are at elevated risk for renal complications. These patients might benefit from continuous glucose monitoring to characterize their glycemic variability, dietary modifications focused on reducing postprandial glucose excursions, or medications that specifically flatten glycemic peaks. Although SHR was independently associated with albuminuria after adjustment for demographic, socioeconomic, lifestyle, biochemical, and comorbidity factors. However, whether SHR improves discrimination, calibration, reclassification, or clinical decision‐making beyond simpler glycemic markers remains unclear and should be addressed in future prospective studies.

### 4.1 Study Limitations

Our study has several limitations. First, the cross‐sectional design precludes any inference of causality. While we hypothesize that SHR‐related metabolic stress leads to renal damage, we cannot exclude the possibility that pre‐existing renal impairment influences glucose metabolism and HbA1c levels. Therefore, the observed association should be interpreted with caution. Longitudinal studies are warranted to establish the temporal relationship and determine whether elevated SHR precedes the development of albuminuria. Second, although we adjusted for a wide array of confounders, residual confounding by medication use cannot be entirely excluded, and future prospective studies with detailed longitudinal medication records are warranted to confirm our findings. Third, SHR and albuminuria in this study were both derived from single measurements, which may not fully reflect long‐term glycemic variability or persistent urinary albumin excretion. Future longitudinal studies with repeated assessments are warranted to validate the observed associations. Finally, the NHANES data, while nationally representative, may not be fully generalizable to other populations with different genetic and environmental backgrounds. Despite these limitations, our study has considerable strengths, including the large, well‐characterized, representative sample, the use of standardized protocols for laboratory measurements, and the comprehensive adjustment for potential confounders.

## 5 Conclusions

In conclusion, this population‐based study provides compelling evidence that an elevated stress hyperglycemia ratio is independently associated with a higher prevalence of albuminuria among US adults with hypertension. This association is nonlinear (J‐shaped), significant at both low and high extremes of SHR, and robust across key subgroups, including individuals without diabetes. These findings suggest that SHR is a readily available and promising biomarker that reflects an aspect of dysglycemia relevant to renal microvascular health that is not captured by HbA1c alone. Assessing SHR could improve risk stratification for renal impairment in hypertensive patients and identify those who might benefit from interventions aimed at stabilizing glycemic control, even in the absence of overt diabetes. Future prospective studies are warranted to confirm the causal nature of this association and to explore whether targeting SHR can effectively prevent the development and progression of renal disease in this population.

## Conflicts of Interest

The authors declare no conflicts of interest.

## Supporting information


**Table S1:** Multicollinearity assessment.

**Table S2:** Logistic regression analysis of the association between SHR and albuminuria.

**Table S3:** Logistic regression analysis of the association between SHR and albuminuria.

**Table S4:** Sensitivity analysis of the association between SHR and albuminuria using the lowest exposure quartile (Q1) as the reference category.

## Data Availability

The data that support the findings of this study are available from the corresponding author upon reasonable request.
