# Supplementary material for: The Association Between Stress‐Induced Hyperglycemia Ratio and Increased Urinary Albumin Excretion in Patients With Hypertension: A Population‐Based Study
Source: Kaohsiung J Med Sci. 2026 May 14:e70237. Online ahead of print. doi: 10.1002/kjm2.70237 (PMC13399739; doi:10.1002/kjm2.70237)
Supplement: Supplementary file 1 — Table S1: Multicollinearity assessment. [file KJM2-9999-e70237-s001.docx]

**Supplementary Table S1. Multicollinearity assessment.**

| **Variable** | VIF |
| --- | --- |
| Age | 2.322 |
| Sex | 1.887 |
| Race | 1.381 |
| BMI | 1.485 |
| Education | 1.459 |
| PIR | 1.426 |
| Smoking status | 1.438 |
| Drinking status | 1.312 |
| Physical activity | 1.402 |
| Marital status | 1.477 |
| Total cholesterol | 1.576 |
| Uric acid | 1.598 |
| Hemoglobin | 1.609 |
| eGFR | 2.232 |
| Diabetes | 1.442 |
| CVD | 1.368 |
| Cancer | 1.561 |

**Abbreviations:** BMI, body mass index; PIR, Poverty income ratio; eGFR, Estimated glomerular filtration rate; CVD, Cardiovascular disease.
