# Supplementary material for: The Association Between Stress‐Induced Hyperglycemia Ratio and Increased Urinary Albumin Excretion in Patients With Hypertension: A Population‐Based Study
Source: Kaohsiung J Med Sci. 2026 May 14:e70237. Online ahead of print. doi: 10.1002/kjm2.70237 (PMC13399739; doi:10.1002/kjm2.70237)
Supplement: Supplementary file 3 — Table S3: Logistic regression analysis of the association between SHR and albuminuria. [file KJM2-9999-e70237-s004.docx]

**Supplementary Table S3. Logistic regression analysis of the association between SHR and albuminuria.**

| **Categories** |  | **Model 1** | | **Model 2** | | **Model 3** | |
| --- | --- | --- | --- | --- | --- | --- | --- |
|  |  | **OR (95% CI)** | ***P-value*** | **OR (95% CI)** | ***P-value*** | **OR (95% CI)** | ***P-value*** |
| **Continuous** | | 2.83 (1.69-4.73) | <0.001 | 4.47 (2.56-7.81) | <0.001 | 3.03 (1.43-6.42) | 0.005 |
| **Categories** |  |  |  |  |  |  |  |
| Quartile 1 |  | 1.60 (1.29-2.00) | <0.001 | 1.46 (1.15-1.85) | 0.002 | 1.53 (1.10-2.12) | 0.012 |
| Quartile 2 |  | Ref |  | Ref |  | Ref |  |
| Quartile 3 |  | 1.26 (0.98-1.60) | 0.068 | 1.38 (1.07-1.76) | 0.012 | 1.46 (1.04-2.07) | 0.032 |
| Quartile 4 |  | 2.03 (1.61-2.54) | <0.001 | 2.29 (1.79-2.91) | <0.001 | 2.23 (1.52-3.26) | <0.001 |
| *P* for trend |  |  | <0.001 |  | <0.001 |  | <0.001 |

**Model 1**: unadjusted.

**Model 2**: adjusted for age, sex, race.

**Model 3**: adjusted for age, sex, race, body mass index, education, poverty income ratio, smoking status, drinking status, physical activity, marital status, triglyceride-glucose index, total cholesterol, uric acid, hemoglobin, estimated glomerular filtration rate, diabetes, cardiovascular disease, and cancer.
