# Supplementary material for: The Association Between Stress‐Induced Hyperglycemia Ratio and Increased Urinary Albumin Excretion in Patients With Hypertension: A Population‐Based Study
Source: Kaohsiung J Med Sci. 2026 May 14:e70237. Online ahead of print. doi: 10.1002/kjm2.70237 (PMC13399739; doi:10.1002/kjm2.70237)
Supplement: Supplementary file 4 — Table S4: Sensitivity analysis of the association between SHR and albuminuria using the lowest exposure quartile (Q1) as the reference category. [file KJM2-9999-e70237-s002.docx]

**Supplementary Table S4.** **Sensitivity analysis of the association between SHR and albuminuria using the lowest exposure quartile (Q1) as the reference category.**

| **Categories** |  | **Model 1** | | **Model 2** | | **Model 3** | |
| --- | --- | --- | --- | --- | --- | --- | --- |
|  |  | **OR (95% CI)** | ***P-value*** | **OR (95% CI)** | ***P-value*** | **OR (95% CI)** | ***P-value*** |
| **Continuous** | | 2.83 (1.69-4.73) | <0.001 | 4.47 (2.56-7.81) | <0.001 | 3.75 (2.06-6.85) | <0.001 |
| **Categories** |  |  |  |  |  |  |  |
| Quartile 1 |  | Ref |  | Ref |  | Ref |  |
| Quartile 2 |  | 0.62 (0.50-0.78) | <0.001 | 0.68 (0.54-0.87) | 0.002 | 0.68 (0.49-0.94) | 0.022 |
| Quartile 3 |  | 0.78 (0.62-0.99) | 0.038 | 0.94 (0.73-1.20) | 0.626 | 1.01 (0.72-1.42) | 0.962 |
| Quartile 4 |  | 1.26 (1.04-1.53) | 0.016 | 1.56 (1.26-1.94) | <0.001 | 1.60 (1.22-2.09) | 0.001 |
| *P* for trend |  |  | <0.001 |  | <0.001 |  | <0.001 |

**Model 1**: unadjusted.

**Model 2**: adjusted for age, sex, race.

**Model 3**: adjusted for age, sex, race, body mass index, education, poverty income ratio, smoking status, drinking status, physical activity, marital status, total cholesterol, uric acid, hemoglobin, estimated glomerular filtration rate, diabetes, cardiovascular disease, and cancer.
